# HCV kinetic and modeling analyses project shorter durations to cure under combined therapy with daclatasvir and asunaprevir in chronic HCV-infected patients

**DOI:** 10.1371/journal.pone.0187409

**Published:** 2017-12-07

**Authors:** Laetitia Canini, Michio Imamura, Yoshiiku Kawakami, Susan L. Uprichard, Scott J. Cotler, Harel Dahari, Kazuaki Chayama

**Affiliations:** 1 The program for Experimental & Theoretical Modeling, Division of Hepatology, Department of Medicine, Loyola University Medical Center, Maywood, Illinois, United States of America; 2 Centre for Immunity, Infection and Evolution, University of Edinburgh, Edinburgh, United Kingdom; 3 Department of Gastroenterology and Metabolism, Applied Life Sciences, Institute of Biomedical & Health Sciences, Hiroshima University, Hiroshima, Japan; Centre de Recherche en Cancerologie de Lyon, FRANCE

## Abstract

**Background & aims:**

High cure rates are achieved in HCV genotype-1b patients treated with daclatasvir and asunaprevir, DCV/ASV. Here we analyzed early HCV kinetics in genotype-1b infected Japanese subjects treated with DCV/ASV and retrospectively projected, using mathematical modeling, whether shorter treatment durations might be effective.

**Methods:**

HCV RNA levels were measured frequently during DCV/ASV therapy in 95 consecutively treated patients at a single center in Japan. Mathematical modeling was used to predict the time to cure, i.e, <1 virus copy in the extracellular body fluid. Patients with HCV<15 IU/ml at week 1 (n = 27) were excluded from modeling analysis due to insufficient HCV RNA data points.

**Results:**

Eighty nine of the 95 included patients (94%) achieved cure, 3 (3%) relapsed due to treatment-emergent resistance, and 3 (3%) completed therapy but were lost during follow up. Model fits from 68 patients with sufficient data points indicate that after a short pharmacological delay (15.4 min [relative standard error, rse = 26%]), DCV/ASV effectiveness in blocking HCV production was 0.999 [rse~0%], HCV half-life in blood was t_1/2_ = 1.7 hr [rse = 21%], and HCV-infected cell loss rate was 0.391/d [rse = 5%]. Modeling predicted that 100% and 98.5% of patients who had HCV<15 IU/ml at days 14 and 28 might have been cured with 6 and 8 weeks of therapy, respectively. There was a trend (p = 0.058) between younger age and shorter time to cure.

**Conclusion:**

Modeling early HCV kinetics under DCV/ASV predicts that most patients would achieve cure with short treatment durations, suggesting that 24 weeks of DCV/ASV treatment can be significantly shortened.

## Introduction

Direct-acting antivirals (DAAs) have transformed treatment for hepatitis C virus (HCV) [1,2]. Japan was the first country to approve interferon-free oral DAA therapy consisting of 24 weeks of asunaprevir, ASV (a 2^nd^ generation HCV NS3 serine protease inhibitor) and daclatasvir, DCV (an HCV NS5A inhibitor). Twenty-four weeks of ASV/DCV was associated with ~90% sustained-virological response (SVR, or cure) rates in genotype-1b patients [3]. While DCV/ASV is not available in the United States, it is widely used in Asia [4]. The high SVR rates with 24 weeks of treatment raise the possibility that cure might be achieved with shorter duration of therapy. Interestingly, several cases of SVR after 2 to 12 weeks of DCV/ASV were reported, confirming that <24 week treatment duration is indeed possible [5,6]. There is an urgent need to reduce the cost and optimize treatment of HCV in Asia [7]. Decreasing duration of DAA therapy would provide cost saving, improve compliance, and could reduce the development of ALT elevations, the most common adverse event reported with DCV/ASV, that effects 5% of patients and begins ~10 weeks after initiation of treatment [4].

On treatment mathematical modeling successfully predicted the duration of IFN-free therapy with silibinin + ribavirin needed to achieve cure [8]. Mathematical modeling has also been applied to data from 58 HCV genotype-1 infected patients who were treated for 12 weeks with three different IFN-free approved sofosbuvir (SOF)-based regimens [9] and predicted that the majority of patients could have been cured with ≤8 weeks of SOF-based therapy. Here we modeled early HCV kinetics in genotype-1b infected patients treated with DCV/ASV and retrospectively projected the duration of therapy needed to achieve cure.

## Methods

### Patients

Ninety-five patients received 24 weeks of daclatasvir (Daklinza, Bristol-Myers) 60 mg daily and asunaprevir (Sunvepra, Bristol-Myers) 200 mg twice daily at a single center in Japan. The mean age was 72±10 years, 37 (39%) were male, 68 (72%) were IFN experienced, mean BMI 23±4, mean platelet count 13.9±6.0 ×10^4^/μL and mean ALT level 38.3±24.0 IU/L. Thirty-three patients (35%) had cirrhosis by either liver biopsy or laboratory algorithm [10]. All subjects gave written informed consent as approved by the hospital ethical committee and conforming to the 1975 Declaration of Helsinki. The study was approved by Hiroshima University ethical committee with approval number DaiEki-1161

### HCV RNA measurements and resistance-associated variants (RAVs)

HCV RNA levels were measured using Roche Cobas Taqman Test v2.0 (CTM) at baseline, at 4, 8, 48 and 72 hours and weeks 1, 4, and 24 during therapy and then 12 weeks after completion. The amino acid sequences of the HCV NS3-D168, NS5A-L31 and -Y93 regions were determined in the 3 relapsers by Invader assay as described in S1 Table. All data are presented in S7 Table.

### Mathematical modeling

HCV viral kinetics under therapy was assumed to follow the standard biphasic model [9]:

$$\frac{dI}{dt}=\beta T_{0}V-\delta I$$ (1)

$$\frac{dV}{dt}=(1-\varepsilon )pI-cV$$

where T_0_ represents the number of target cells (i.e., hepatocytes), I, the number of infected cells and V, is the viral load in blood. Virus, V, infects target cells with rate constant β, generating productively-infected cells, I, which produce new virions at rate p per infected cell. Infected cells are lost at a rate δ per infected cell and virions are assumed to be cleared from blood at rate c per virion. Similar to previous modeling efforts [9], we assumed the target cell level remained constant during therapy at pre-treatment level T_0_ = cδ/βp. DAA effect ε is defined as the therapy effectiveness 0≤ε≤1 in preventing viral production/secretion. We assume that therapy was effective after a pharmacological delay τ. The associations among age, cirrhosis and previous IFN therapy and model parameters were examined with the Wald test as described in S1 Information. Parameter estimates and their inter-individual variability (IIV) estimates were obtained using a maximum-likelihood method by the stochastic approximation expectation-maximization (SAEM) algorithm implemented in MONOLIX 2016R1 (Lixoft, Antony, France). Further details are given in the S1 Information.

### Cure boundaries

The time to cure was defined as the time to reach less than one HCV particle in the entire extracellular body fluid (blood, interstitial and transcellular) volume approximately 13.5L [9]. A value of 7x10^-5^ for *V* (IU/ml) was used as the threshold for cure. A sensitivity analysis was performed assuming 5 L to 20 L of extracellular body fluid volume corresponding to cure threshold values of 2x10^-4^ and 5x10^-5^ IU/mL, respectively.

### Statistical analysis

To study the association between a categorical and a continuous variables we performed an exact trend permutation tests with 1000 Monte Carlo permutations as described in the S1 Information. To test the association between two categorical covariates we performed Fisher’s exact test. For all analyses, a P-value, P≤0.05 was considered as statistically significant. Data analyses were performed using R 3.1.2.

## Results

### Viral kinetics and SVR rates

**Fig 1:**
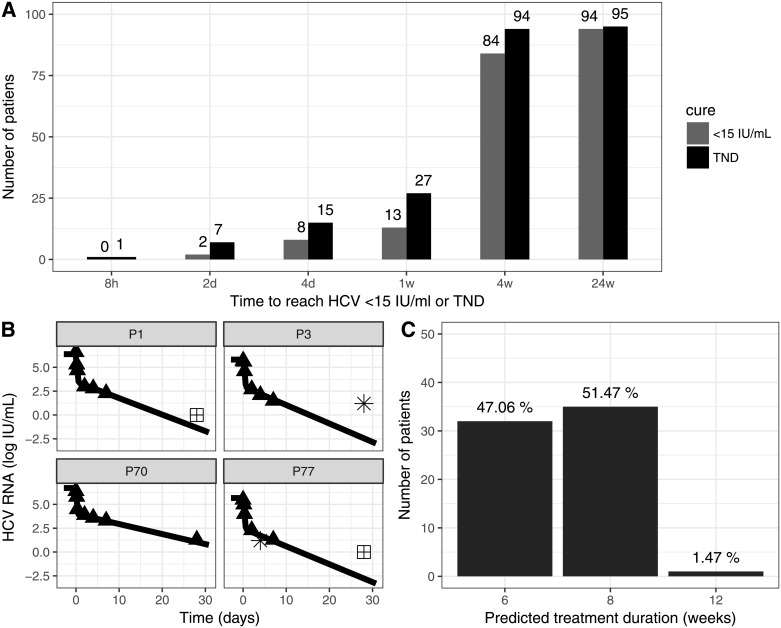
Viral kinetics, model fits and projected time to cure. **(A)** Time (days) to reach HCV <15 IU/ml or target not detected, TND, during therapy. At end of treatment (w24) all patients but one were TND. **(B)** Observed viral kinetics and model curves in 4 representative patients (P). Filled triangles: observed HCV viral load above the limit of quantification, LOQ (>15 IU/mL); stars, observed HCV < 15 IU/mL but still detected; crossed squares, TND (arbitrary set to 1 IU/mL); solid lines, biphasic model (Eq 1) best fit curves (see S3 Table for individual parameters). HCV viral load and fit curves of the remaining subjects are shown in S1 Fig. **(C)** Predicted treatment duration (weeks) to reach cure based on a viral cure defined as <1 virus copy in entire patient extracellular fluid (~13.5L).

Eighty nine of the 95 included patients (94%) achieved SVR, 3 (3%) relapsed due to treatment-emergent NS5A-Y93 and NS3-D168 RAVs (S1 Table), and 3 (3%) completed therapy but were lost during follow up. During therapy, the viral load was <15 IU/mL in 1 patient (1%) after 8hr, in 5 patients (5%) at day 2, in 6 (7%) at day 4, 15 (16%) at week 1, and 64 (70%) at week 4 (Fig 1A). All patients but one had target not detected (TND) at the end of treatment (EOT). The EOT positive patient achieved SVR. Time to HCV<15 IU/ml was not associated with cirrhosis or with relapse.

### Viral kinetic parameter estimation

Due to insufficient data points we excluded 27 patients in whom HCV was <15 IU/ml (detectable or TND) during the first week of treatment (Fig 1A). In total, 68 patients were included in the viral kinetic modeling. The model fit the measured data well (Fig 1B and S1 Fig), the parameters were accurately estimated (S2 and S3 Tables) and the goodness-of-fit plots were satisfactory (S2 Fig). The initial HCV viral load was estimated at 6.05 log_10_ IU/mL (relative standard error, rse = 1%), with an IIV of 8% (rse = 8%). After a pharmacological delay of τ = 15.4 min (rse = 26%) with an IIV of 123% (rse = 14%), DCV/ASV effectiveness in blocking viral production was estimated as ε = 0.999 (rse~0) with an IIV of 0.5% (rse = 22%). Virus clearance rate was estimated as c = 9.73 d^-1^ (rse = 21%) with an IIV of 7% (rse = 87%), leading to virus serum half-life of 1.7 hr. Infected cells loss rate was estimated δ = 0.391 d^-1^ (rse = 5%) with a fixed IIV = 20%, corresponding to an HCV-infected cell half-life of 1.77 days. Viral-kinetic parameters were not associated with previous IFN-based treatment or cirrhosis. We found that virus clearance rate, c, was inversely associated with age (0.038) (S3 Table).

### Predicting time to cure

The mean predicted time to virus clearance from the extracellular body fluid was 6.50±0.95 weeks. To be conservative, we stratified the duration of therapy needed to achieve virus eradication based on model predictions as follows: (i) subjects with predicted viral eradication in less than 6 weeks could be assigned to 6 weeks therapy, (ii) subjects with predicted viral eradication between 6 to 8 weeks could be assigned to 8 weeks therapy, (iii) subjects with predicted viral eradication between 8 to 10 weeks could be assigned to 10 weeks therapy and (iv) subjects with predicted viral eradication between 10 to 12 weeks could be assigned to 12 weeks therapy. 47% (32/68) of patients were predicted to reach <1 virus copy in 13.5 L of extracellular fluid volume within 6 weeks of therapy, 51% (35/68) by 8 weeks, and 1% (1/68) were projected to need the full 12 weeks of therapy (Fig 1C). Thus, modeling predicted that 100% and 98.5% of patients who had HCV <15 IU/ml at days 14 (54/68) and 28 (67/68) might have been cured with 6 and 8 weeks of therapy, respectively. Assuming 5 L of extracellular fluid volume (i.e., higher cure boundary level compared to 13.5 L of extracellular fluid volume), the model predicts that 62% (42/68) of patients were cured within 6 weeks of therapy, 37% (25/68) by 8 weeks, and 1% (1/68) by 12 weeks of therapy (S4 Fig). Assuming 20 L of extracellular fluid volume (i.e., lower cure boundary level compared to 13.5 L of extracellular fluid volume), 38% (26/68) of patients were projected to reach cure within 6 weeks of therapy, 60% (41/68) by 8 weeks, and 1% (1/68) by 12 weeks of therapy (S4 Fig). The predicted time to cure was not significantly different based on cirrhosis status (p = 1.0, Fisher’s exact test), age (p = 0.18, Spearman’s rank correlation test) or previous IFN treatment (p = 0.14, Fisher’s exact test).

Of the three patients who relapsed due to treatment-emergent RAVs (S1 Table), one was TND at week 1 and was not included in the modeling and the other two had a projected time to cure of 10 weeks of treatment. There was no difference in viral kinetics between the 3 patients with treatment-emergent RAVs and those who achieved SVR (S3 Fig). Modeling results did not predict the relapse observed in 3 patients. A more speculative fit analysis of the remaining 27 patients with insufficient data points is shown in the S1 Information, S5 and S6 Tables and S4 Fig.

## Discussion

Modeling of early HCV genotype 1b kinetics during dual therapy with DCV/ASV predicts that all patients would achieve cure with short treatment durations (6–12 weeks), suggesting that 24 weeks of DCV/ASV might be shortened substantially in many patients. Moreover, the modeling predicted that 100% patients who had HCV<15 IU/ml at day 14 and 98.5% with HCV<15 at day 28 might have been cured with 6 and 8 weeks of therapy, respectively. If validated prospectively, the modeling approach used here could be applied in real time to optimize duration of therapy, addressing in part an urgent need to improve treatment for hepatitis C in Asia [7].

Viral-kinetic parameters estimated here were not associated with previous IFN-based treatment or cirrhosis in agreement with a modeling viral kinetic study in patients treated with IFN-free approved SOF-based therapies [9]. The association found in the current study between younger age and higher virus clearance rate (p = 0.038, S2 Table) translated into a trend between younger age and shorter time to cure (p = 0.058, correlation coefficient: 0.23). In patients with advanced cirrhosis we previously reported that the median time to cure was significantly longer in patients with Child-Pugh≥B-7 compared to those with Child-Pugh <B-7 (9.1 weeks [range 4.7–20.2] vs 7.5 weeks [range 2.5–17.3], p = 0.002) and in patients with albumin level, ALB<35 compared to those with ALB≥35 (9.2 weeks [range 4.2–15.1] vs 7.9 [range 2.5–20.2] weeks, p = 0.048) [11]. However, patients with decompensated cirrhosis were not included in the current study.

Initial clinical trials with dual ASV/DCV therapy reported high rates of SVR in patients with genotype 1b infection, but reduced efficacy against genotype 1a [12]. As a result, dual therapy was approved only against genotype 1b as performed in the current study. In order to overcome treatment-emergent RAVs in patients infected with HCV genotype 1a, four DAAs (beclabuvir, sofosbuvir and DCV/ASV) were given in the FOURward study [13] for 4 (n = 14) and 6 (n = 14) weeks of therapy in which ~80% of patients were infected with genotype 1a. The fact that 29% and 57% of patients achieved SVR in the 4 and 6 week regimens, respectively, might indicate that modeling on treatment in genotype 1a patients treated with four DAAs has the potential to predict who will reach cure under longer duration of therapy. Recently, Toyota et al [14], reported that 12 weeks of DCV/ASV in combination with beclabuvir (a non-nucleoside NS5B inhibitor) therapy yielded similar SVR rates (96%) as with 24 week DCV/ASV therapy. Further studies are needed to evaluate whether mathematical modeling can be used to refine treatment duration with this new triple DAA regimen.

Only 3 (3%) patients in the present study did not achieve SVR due to treatment-emergent RAVs (S1 Table). Detailed data describing the kinetics of RAVs were not available and therefore it was not feasible to perform modeling as previously reported [15]. Thus, not unexpectedly the modeling (Eq 1) is unable to predict relapse due to RAVs, but they can be identified directly from post-treatment blood samples.

One patient in the current study, which used the CTM assay to measure HCV RNA, had HCV TND at week 4 and HCV detected at the EOT yet achieved SVR. Since it is not known when HCV RNA became detected again (i.e., between weeks 4 and 24), modeling was only performed in this patient until week 4. Notably, while HCV RNA positive at the end of treatment (EOT+) with IFN-based therapies was an indicator of treatment failure, we and others [16,17] have provided viral kinetic analysis of numerous CHC patients treated with SOF-based therapies, who were HCV RNA detectable at the EOT, yet went on to achieve SVR, termed EOT+/SVR [16,17]. Comparing the HCV RNA detection assays used in these studies and other, we have observed that the Abbott RealTime HCV assay (ART) is able to detect HCV RNA on treatment several weeks longer than CTM and is thus associated with the detection of EOT+/SVR. Consistent with this, recent reports document that other patients treated with IFN-free SOF-based regimens exhibiting EOT+/SVR were observed using the ART assay (reviewed in [18]). Two hypotheses have been offered for the phenomenon of EOT+/SVR, an immunologic mediated clearance that occurs after treatment is completed and/or that DAAs (such as HCV NS5A inhibitors) promote the production of non-infectious viral particles [19]. To explain the phenomenon of EOT+/SVR observed under the more sensitive ART assay, new models are being developed to test these different hypotheses [20,21]. Relevant to this study, the observation that the EOT+/SVR case reported here achieved SVR suggests that EOT positive under DCV/ASV likewise does not equal treatment failure.

In summary, modeling results suggest that DCV/ASV for HCV genotype 1b might be shortened from 24 to 12 weeks without compromising SVR. Moreover, real-time application of viral kinetic analysis has the potential to individualize treatment duration and reduce adverse effects and cost. Prospective on treatment modeling studies with dual and more potent DAA regimens [13,14] are needed to confirm these results.

## Supporting information

S1 Information Description of the nonlinear mixed effect models and parameter estimation and statistical methods. (DOCX)

S1 Table Resistance-associated variants (RAVs). (DOCX)

S2 Table Population parameter estimates. (DOCX)

S3 Table Best individual model parameter estimates. (DOCX)

S4 Table Covariate analysis. (DOCX)

S5 Table Speculative population parameter estimates of the 27 patients with insufficient data points. (DOCX)

S6 Table Speculative model parameter estimates of the 27 patients with insufficient data points. (DOCX)

S7 Table Viral kinetics data. ID: patient identification number; time: time from treatment initiation; DV: dependent variable, i.e. HCV viral load; cens: data below the limit of detection (cens = 1) or above the limit of detection (cens = 0); cirrhosis.x and cirrhosis.y status (0/1); age in years; gender (F/M); weight in kg; height in cm; bmi in kg/m^2^; PLT: platelet count (in x10^4^/μL); ALT: alanine aminotransferase levels (in IU/L); hcv_vira_load.0.: HCV viral load at time = 0; hcv_vira_load.4h.: HCV viral load at time = 4h; hcv_vira_load.8h.: HCV viral load at time = 8h; hcv_vira_load.48h.: HCV viral load at time = 48h; hcv_vira_load.96h.: HCV viral load at time = 96h; hcv_vira_load.1w.: HCV viral load at time = 1 week; hcv_vira_load.4w.: HCV viral load at time = 4 weeks; hcv_vira_load.24w.: HCV viral load at time = 24 weeks; hcv_vira_load.post24w.: HCV viral load at time >24 weeks; core70.91=; DCV.resistance..L31.Y93.: resistance to daclatasvir—mutation at sites L31 and Y93; ASV.resistance: resistance to asunaprevir; biopsy: METAVIR scores; Genotype: HCV genotype; previous.IFN: use of IFN therapy previously to this study (yes/nothing); IFN: if previous.IFN = yes, type of IFN used for therapy previously to this study.
(TXT)

S1 Fig Individual model fits. Best model fit curves are shown with black lines. Each box represent a patient (patient number in the strip above each box). The observed viral titer are represented by the dots. Data above the HCV RNA limit of quantification are shown in pink and data below the limit of detection in blue. HCV detected but not quantified is shown in green.
(DOCX)

S2 Fig Goodness of fit plots. A) Visual predictive check represents the empirical percentiles (10%, 50% and 90%, green lines) and the 90% confidence intervals for these percentiles computed from 500 simulations of the observations based on the model, according to the original study design. B) Residuals plots. Observations are shown by dots and BLQ data are in red. The upper panels show population weighted residuals (PWRES) (left panel) and individual weighted residuals (IWRES) (left panels) depending on time. The lower panels PWRES (left panel) and IWRES (right panels) depending on predictions.
(DOCX)

S3 Fig Viral kinetics in patients (n = 3) with treatment- emergent resistance-associated variants, RAVs (colored lines) and without RAVs (grey lines). Patients with pre-treatment NS5A Y93H RAVs (Pt 75; blue line) and without (Pts 65 and 79; red curves) as described in S1 Table. Observations below the lower limit of quantification (LLOQ = 15 IU/mL) or not detected (TND) are shown with triangles whereas those above this limit are shown by circles.
(DOCX)

S4 Fig Repartition of the predicted duration of treatment to achieve virus cure for patients with TND before 1 week (in blue) and after 1 week (in red). (DOCX)
