# Supplementary material for: HCV kinetic and modeling analyses project shorter durations to cure under combined therapy with daclatasvir and asunaprevir in chronic HCV-infected patients
Source: PLoS One. 2017 Dec 7;12(12):e0187409. doi: 10.1371/journal.pone.0187409 (PMC5720697; doi:10.1371/journal.pone.0187409)
Supplement: S1 Information — (DOCX) [file pone.0187409.s001.docx]

**S1 Information**

**(1) Description of the nonlinear mixed effect models.**

Nonlinear mixed effect models (or population approach) were first developed to study the pharmacokinetics (PK) of drugs [1]. This method allows a description of population characteristics (fixed effect) as well as the inter-individual variability (IIV, i.e. random effect) [2]. In this method, a function *f* describing the variables being modeled, e.g., the viral load, depends nonlinearly on *θ_i_*, a vector of the *p* parameters of subject *i*. A vector *ξ_i_* representing the times at which samples are collected from subject *i*, *ξ_i_ = (t_i1_; t_i2_;…; t_in_)*, is also considered. The statistical model for subject *i* is then given by:

$$y_{i}=f\left(\theta_{i};\xi_{i}\right)+ae_{i}$$

where *y_i_* is a vector with *n_i_* observations of subject *i*, with *i* varying from 1 to *N*, *ε_i_* is the vector of the residual errors which is the part of the observations unexplained by the model *f*. It is assumed that the errors *ε_i_* are independent from one observation to another and that their distribution is Gaussian *ε_i_ ~ N(0; I_ni_)*, where *I_ni_* is an identity matrix of dimension *n_i_*. *a* is a parameter characterizing the error model variance.

In nonlinear mixed effect models, the model *f* is common to all the subjects, but the vector of parameters *θ_i_* for subject *i* may vary from one subject to another. The inter-individual variability is modeled with the vector of random effect parameters *η_i_*. The vector of parameters *θ_i_* for the subject *i* can then be expressed as a second-level model which links with the function g, the vector of fixed effect parameters *β* common for all subject and the vector of random effects *η_i_* specific for subject i: *θ_i_* = g(β;*η_i_*). The vector of random effect is assumed to follow a Gaussian distribution *η_i_ ~ N(0; Ω)*, *η_i_* and *ε_i_* are assumed to be independent for subject *i* and *η_i_* |*ε_i_* is assumed independent from one subject to another. *Ω* is the matrix of random effect variance. Here, the function *g* is an exponential model. The vector of parameters is hence written as $\theta_{i}=\beta e^{\eta_{i}}$.

The residual error measures the difference between predictions and observations. The model used was: $y=f+a\varepsilon$. *y* is the observed response, *f* is the model function, *a* is the additive error term, and the error *ε* is normally distributed following $N(0,\sigma^{2})$ where $\sigma^{2}$is the variance of the residual variability.

**(2) Parameter estimation and statistical methods.**

HCV RNA data including the first viral load below the limit of quantification (<15 IU/ml) or below the limit of detection was used for model fits, using a population approach, whereas the subsequent observations were truncated. The population parameters and their inter-individual variability (IIV) estimates were obtained using a maximum-likelihood method implemented in MONOLIX 2016R1 (Lixoft, Antony, France), which uses the stochastic approximation expectation-approximation (SAEM) algorithm [3] to estimate population parameters. The model was fit to log10 viral load. Individual parameters were estimated using the empirical Bayes method [4]. Permutation tests were performed with 1000 Monte Carlo permutations. We used the “perm” package implemented in R [5].

**(3) A speculative analysis of the 27 patients with insufficient data points for modeling fitting.**

We sought to predict the time to cure in the remaining 27 patients who had HCV negativity within the first week of therapy. To be conservative we assumed that the 27 patients had the lowest estimated individual infected cell loss rate constant (δ=0.243/day) estimated from the 68 patients with sufficient data points for modeling (S1 and S2 Tables). A significantly (P=0.002) lower baseline viral load (5.17±1.00 log_10_ IU/mL) compared to the other 68 patients (6.06±0.55 log_10_ IU/mL), was found (S5 and S6 Tables). The analysis suggests that 1 patient might have been cured with 6 weeks of therapy, 10 with 8 weeks and 16 with 10 weeks of therapy (S3 Fig.).

**References:**

[1] Sheiner LB, Steimer JL. Pharmacokinetic/pharmacodynamic modeling in drug development. Annu Rev Pharmacol Toxicol 2000;40:67-95.

[2] Karlsson MO, Sheiner LB. The importance of modeling interoccasion variability in population pharmacokinetic analyses. J Pharmacokinet Biopharm 1993;21:735-750.

[3] Kuhn E, Lavielle M. Maximum likelihood estimation in nonlinear mixed effects models. Computational Statistics & Data Analysis 2005;49:1020-1038.

[4] Pinheiro J, Bates D. Mixed-effects models in S and S-PLUS. New York: Springer Verlag, 2000.

[5] Fay MP, Shaw PA. Exact and Asymptotic Weighted Logrank Tests for Interval Censored Data: The interval R package. J Stat Softw 2010;36.
