# Supplementary material for: HCV kinetic and modeling analyses project shorter durations to cure under combined therapy with daclatasvir and asunaprevir in chronic HCV-infected patients
Source: PLoS One. 2017 Dec 7;12(12):e0187409. doi: 10.1371/journal.pone.0187409 (PMC5720697; doi:10.1371/journal.pone.0187409)
Supplement: S1 Table — (DOCX) [file pone.0187409.s002.docx]

**S1 Table. Resistance-associated variants (RAVs)**

|  |  |  | **Pre treatment** | | | |  | **Post treatment** | | |
| --- | --- | --- | --- | --- | --- | --- | --- | --- | --- | --- |
| Pt | Age | Sex | CH  /LC | NS3  -D168 | NS5A  -L31 | NS5A  -Y93 | outcome | NS3  -D168 | NS5A  -L31 | NS5A  -Y93 |
| 42 | 65 | M | CH | D | L | Y | Relapse | V (25-75%) | H (25-75%) | H (99%<) |
| 75 | 74 | M | LC | D | L | H (25-75%) | Relapse | V (90-99%) | L | H (99%<) |
| 62 | 79 | M | CH | D | L | Y | Relapse* | E (99%<) | L | H (99%<) |

The amino acid sequences were determined by Invader assay as previously reported [1]. The lower detectable limit for the population frequency of NS3 and NS5A drug resistance-associated variants (RAVs) was approximately 1%. * Treatment discontinuation at 21 weeks due to neghroric syndrome.

Reference:

[1] Yoshimi S, Ochi H, Murakami E, Uchida T, Kan H, Akamatsu S, Hayes CN, et al. Rapid, Sensitive, and Accurate Evaluation of Drug Resistant Mutant (NS5A-Y93H) Strain Frequency in Genotype 1b HCV by Invader Assay. PLoS One 2015;10:e0130022.
