# Supplementary material for: HCV kinetic and modeling analyses project shorter durations to cure under combined therapy with daclatasvir and asunaprevir in chronic HCV-infected patients
Source: PLoS One. 2017 Dec 7;12(12):e0187409. doi: 10.1371/journal.pone.0187409 (PMC5720697; doi:10.1371/journal.pone.0187409)
Supplement: S2 Table — (DOCX) [file pone.0187409.s003.docx]

**S2 Table.** Population parameter estimates

| **Parameter type** | ***ε*** | ***V_0_***  **(log_10_ IU/mL)** | ***δ***  **(d^-1^)** | ***c***  **(d^−1^)** | ***τ***  **(min)** |
| --- | --- | --- | --- | --- | --- |
| Estimates  (rse %) | 0.999  (~0) | 6.03  (1) | 0.391  (5) | 9.73  (21)  *γ*=0.00596  (48) | 15.4  (26) |
| IIV %  (rse %) | 0.05  (22) | 8  (8) | 20  (-) | 7.3  (87) | 123  (14) |

*ε*: treatment effectiveness in blocking viral production; *V_0_*: baseline HCV RNA; *δ*: infected-cell loss rate; *c*: virus clearance rate; *τ*: pharmacological delay; rse: relative standard error; IIV: Inter-individual variability. Additive error: *a*=0.259 (rse=4%). *γ* parameter represents the estimated association between age and parameter *c*. P-value of the Wald test for the null hypothesis *γ*=0: P-value=0.038. Individual clearance rate is given for patient i by: $c_{i}=c_{pop}\times e^{\gamma\times age}\times e^{\eta_{i}}$ where c=c_pop­_ is the population parameter and η_i_ the individual random effect.
