# Supplementary material for: HCV kinetic and modeling analyses project shorter durations to cure under combined therapy with daclatasvir and asunaprevir in chronic HCV-infected patients
Source: PLoS One. 2017 Dec 7;12(12):e0187409. doi: 10.1371/journal.pone.0187409 (PMC5720697; doi:10.1371/journal.pone.0187409)
Supplement: S3 Table — (DOCX) [file pone.0187409.s004.docx]

**S3 Table:** Best individual model parameter estimates.

| **Patient**  **ID** | **δ**  **(d^-1^)** | **c**  **(d^-1^)** | **ε**  **(d^-1^)** | **τ**  **(min)** | **V0**  **(log_10_ IU/mL)** | **cirrhosis** | **age** | **predicted time to cure**  **(weeks)** |
| --- | --- | --- | --- | --- | --- | --- | --- | --- |
| 1 | 0.40 | 13.59 | 0.999 | 15.47 | 6.38 | 0 | 58 | 8 |
| 3 | 0.45 | 13.94 | 0.999 | 171.42 | 5.81 | 1 | 63 | 6 |
| 4 | 0.38 | 14.12 | 0.998 | 39.00 | 6.15 | 0 | 67 | 8 |
| 5 | 0.43 | 14.70 | 0.999 | 113.44 | 6.10 | 0 | 73 | 6 |
| 6 | 0.40 | 14.59 | 0.999 | 39.11 | 6.56 | 0 | 73 | 8 |
| 7 | 0.44 | 14.17 | 0.999 | 12.11 | 5.96 | 0 | 62 | 6 |
| 10 | 0.37 | 16.74 | 0.998 | 10.68 | 5.66 | 1 | 84 | 8 |
| 12 | 0.38 | 14.97 | 0.999 | 12.06 | 6.16 | 0 | 66 | 8 |
| 13 | 0.35 | 13.15 | 0.998 | 11.92 | 6.34 | 1 | 52 | 8 |
| 14 | 0.38 | 16.33 | 0.999 | 14.04 | 6.29 | 0 | 85 | 8 |
| 15 | 0.35 | 16.44 | 0.998 | 11.10 | 5.55 | 1 | 83 | 8 |
| 16 | 0.40 | 14.32 | 0.999 | 15.41 | 6.21 | 0 | 63 | 8 |
| 18 | 0.39 | 14.69 | 0.999 | 12.81 | 6.19 | 0 | 66 | 8 |
| 19 | 0.35 | 15.74 | 0.998 | 11.12 | 6.28 | 0 | 75 | 8 |
| 21 | 0.46 | 14.93 | 0.999 | 15.47 | 6.40 | 0 | 74 | 6 |
| 22*** | 0.33 | 13.95 | 0.998 | 10.72 | 5.46 | 1 | 55 | 8 |
| 23 | 0.44 | 15.17 | 0.999 | 14.73 | 5.83 | 0 | 75 | 6 |
| 26 | 0.33 | 15.22 | 0.998 | 13.40 | 6.03 | 0 | 78 | 8 |
| 27 | 0.41 | 15.25 | 0.999 | 15.97 | 6.30 | 0 | 76 | 8 |
| 28 | 0.39 | 15.01 | 0.999 | 15.48 | 6.39 | 1 | 73 | 8 |
| 30 | 0.39 | 14.30 | 0.999 | 12.39 | 6.02 | 0 | 61 | 8 |
| 31 | 0.43 | 13.67 | 0.999 | 14.70 | 6.22 | 0 | 56 | 6 |
| 32 | 0.39 | 14.70 | 0.999 | 15.65 | 5.77 | 0 | 72 | 8 |
| 33 | 0.37 | 14.67 | 0.999 | 14.06 | 5.31 | 1 | 70 | 8 |
| 34 | 0.42 | 15.91 | 0.999 | 11.58 | 5.97 | 0 | 77 | 6 |
| 36 | 0.49 | 13.58 | 0.999 | 21.54 | 6.57 | 0 | 57 | 6 |
| 37 | 0.44 | 14.27 | 0.999 | 12.11 | 5.76 | 0 | 59 | 6 |
| 38 | 0.43 | 15.40 | 0.999 | 14.96 | 6.49 | 0 | 78 | 8 |
| 39** | 0.45 | 14.91 | 0.999 | 25.94 | 5.88 | 0 | 77 | 6 |
| 41 | 0.48 | 15.28 | 0.999 | 13.51 | 6.40 | 0 | 75 | 6 |
| 43 | 0.33 | 15.37 | 0.998 | 11.64 | 5.70 | 1 | 75 | 8 |
| 47 | 0.37 | 14.80 | 0.998 | 12.11 | 6.01 | 0 | 68 | 8 |
| 48 | 0.34 | 15.84 | 0.998 | 10.69 | 6.24 | 0 | 75 | 8 |
| 49 | 0.37 | 14.73 | 0.999 | 16.79 | 6.08 | 0 | 74 | 8 |
| 51 | 0.37 | 15.92 | 0.998 | 13.58 | 5.27 | 0 | 83 | 6 |
| 52 | 0.49 | 14.41 | 0.999 | 13.82 | 6.29 | 0 | 66 | 6 |
| 53 | 0.44 | 14.04 | 0.999 | 62.81 | 5.83 | 1 | 67 | 8 |
| 54 | 0.34 | 16.13 | 0.998 | 15.19 | 5.89 | 0 | 82 | 8 |
| 55 | 0.34 | 15.92 | 0.998 | 9.01 | 6.22 | 0 | 72 | 8 |
| 56 | 0.39 | 14.43 | 0.998 | 11.61 | 5.46 | 0 | 61 | 8 |
| 58 | 0.37 | 15.26 | 0.998 | 77.14 | 6.27 | 1 | 78 | 6 |
| 59 | 0.44 | 15.38 | 0.999 | 12.60 | 5.77 | 0 | 75 | 8 |
| 60 | 0.39 | 15.23 | 0.998 | 16.00 | 6.89 | 0 | 79 | 8 |
| 61 | 0.37 | 14.81 | 0.998 | 13.07 | 5.60 | 1 | 69 | 6 |
| 62* | 0.41 | 15.55 | 0.999 | 15.74 | 5.67 | 0 | 79 | 8 |
| 63 | 0.38 | 14.42 | 0.998 | 12.10 | 5.85 | 1 | 65 | 8 |
| 65 | 0.42 | 15.69 | 0.999 | 17.53 | 6.44 | 0 | 82 | 8 |
| 66 | 0.43 | 14.50 | 0.999 | 34.92 | 6.41 | 1 | 72 | 8 |
| 67 | 0.43 | 14.18 | 0.999 | 57.48 | 6.80 | 0 | 67 | 6 |
| 68 | 0.42 | 15.10 | 1.000 | 146.55 | 6.64 | 1 | 79 | 8 |
| 69 | 0.38 | 14.74 | 0.999 | 17.43 | 6.28 | 1 | 74 | 12 |
| 70 | 0.24 | 15.68 | 0.998 | 12.95 | 6.72 | 0 | 79 | 6 |
| 72 | 0.38 | 14.57 | 0.999 | 12.22 | 5.15 | 0 | 69 | 8 |
| 73 | 0.41 | 16.10 | 0.999 | 31.94 | 6.55 | 0 | 88 | 6 |
| 74 | 0.44 | 14.89 | 0.999 | 20.17 | 6.04 | 1 | 73 | 8 |
| 75* | 0.42 | 14.97 | 0.998 | 15.07 | 6.10 | 1 | 74 | 8 |
| 76 | 0.38 | 14.38 | 0.998 | 12.56 | 6.03 | 0 | 69 | 6 |
| 77 | 0.43 | 13.51 | 0.999 | 28.91 | 5.66 | 1 | 59 | 8 |
| 79 | 0.41 | 15.05 | 0.998 | 225.19 | 6.59 | 1 | 78 | 8 |
| 80 | 0.42 | 14.77 | 0.999 | 13.88 | 6.05 | 0 | 70 | 6 |
| 87 | 0.49 | 14.89 | 0.999 | 13.29 | 6.03 | 1 | 74 | 8 |
| 88 | 0.38 | 14.14 | 0.999 | 21.26 | 6.34 | 1 | 68 | 8 |
| 89 | 0.40 | 13.69 | 0.999 | 28.28 | 6.01 | 1 | 61 | 8 |
| 90*** | 0.27 | 17.68 | 0.999 | 6.25 | 4.27 | 0 | 79 | 8 |
| 91 | 0.39 | 14.36 | 0.999 | 13.70 | 5.70 | 0 | 65 | 8 |
| 93 | 0.40 | 16.73 | 0.999 | 13.84 | 6.09 | 0 | 88 | 8 |
| 94*** | 0.45 | 14.30 | 0.999 | 10.71 | 6.09 | 1 | 57 | 6 |
| 95 | 0.36 | 15.74 | 0.998 | 12.80 | 6.74 | 1 | 82 | 8 |

V_0_, baseline HCV RNA; δ, infected-cell loss rate; ε, therapy effectiveness; c, virus clearance; *Relapsers; **End of treatment positive; *** Lost during follow up. Twenty seven patients were excluded due to insufficient data points for fitting. However, a speculative modeling approach (based on the fitting results shown in this table) in the 27 subjects is shown below (S5 and S6 Tables; S3 Fig.).
