# Supplementary material for: HCV kinetic and modeling analyses project shorter durations to cure under combined therapy with daclatasvir and asunaprevir in chronic HCV-infected patients
Source: PLoS One. 2017 Dec 7;12(12):e0187409. doi: 10.1371/journal.pone.0187409 (PMC5720697; doi:10.1371/journal.pone.0187409)
Supplement: S4 Table — (DOCX) [file pone.0187409.s005.docx]

**S4 Table: Covariate analysis**

| **Model** | **p-value** | **BIC** |
| --- | --- | --- |
| No covariate | - | 376 |
| **τ x cirrhosis** | **0.00059** | **369** |
| **c x cirrhosis** | **0.013** | **366** |
| ε x cirrhosis | 0.11 | 370 |
| V0 x cirrhosis | 0.72 | 379 |
| (**τ+c**) x cirrhosis | 0.0038 for (τ x cirrhosis) and 0.73 for (c x age) | 376 |
| τ x IFN | 0.96 | 366 |
| c x IFN | 0.064 | 351 |
| ε x IFN | 0.51 | 379 |
| V0 x IFN | 0.14 | 373 |
| τ x age | 0.51 | 360 |
| **c x age** | **0.038** | **355** |
| ε x age | 0.20 | 362 |
| V0 x age | 0.82 | 360 |
| c x(age + cirrhosis) | 0.056 for cirrhosis and 0.042 for age | 384 |
| c x age + τ x cirrhosis | 0.051 (for c x age) and 0.0038 (for t x cirrhosis) | 359 |

p-value computed from a Wald test; BIC: Bayesian information criteria. Bold: model with significant association between covariate and parameter. Underlined: selected model according to BIC using the rule “the smaller the better”. *ε*: treatment effectiveness in blocking viral production; *V_0_*: baseline HCV RNA; *δ*: infected-cell loss rate; *c*: virus clearance rate; *τ*: pharmacological delay; IFN, (peg)interferon-alpha-treatment experience.
