# Supplementary material for: HCV kinetic and modeling analyses project shorter durations to cure under combined therapy with daclatasvir and asunaprevir in chronic HCV-infected patients
Source: PLoS One. 2017 Dec 7;12(12):e0187409. doi: 10.1371/journal.pone.0187409 (PMC5720697; doi:10.1371/journal.pone.0187409)
Supplement: S5 Table — (DOCX) [file pone.0187409.s006.docx]

| **Parameter type** | ***ε*** | ***V_0_***  **(log_10_ IU/mL)** | ***δ***  **(d^-1^)** | ***c***  **(d^−1^)** | ***τ***  **(min)** |
| --- | --- | --- | --- | --- | --- |
| Estimates  (rse %) | ~ 1.000  (~0) | 4.45  (4) | 0.243  (FIXED) | 3.59  (7) | 0  (FIXED) |
| IIV %  (rse %) | - | 19  (13) | - | - | - |

**S5 Table.** Speculative population parameter estimates of the 27 patients with insufficient data points. Paramters as defined in S2 Table. To avoid identifiability issues, τ was assumed 0. ε<1.
