# Supplementary material for: HCV kinetic and modeling analyses project shorter durations to cure under combined therapy with daclatasvir and asunaprevir in chronic HCV-infected patients
Source: PLoS One. 2017 Dec 7;12(12):e0187409. doi: 10.1371/journal.pone.0187409 (PMC5720697; doi:10.1371/journal.pone.0187409)
Supplement: S6 Table — (DOCX) [file pone.0187409.s007.docx]

**S6 Table:** Speculative model parameter estimates of the 27 patients with insufficient data points

| **Patient**  **ID** | **δ**  **(d^-1^)** | **c**  **(d^-1^)** | **ε**  **(d^-1^)** | **τ**  **(min)** | **V0**  **(log_10_ IU/mL)** | **cirrhosis** | **age** | **predicted time to cure**  **(weeks)** |
| --- | --- | --- | --- | --- | --- | --- | --- | --- |
| 2 | 0.243 | 3.59 | ~1.000 | 0 | 6.18 | 1 | 81 | 10 |
| 8 | 0.243 | 3.59 | ~1.000 | 0 | 2.92 | 0 | 84 | 6 |
| 9 | 0.243 | 3.59 | ~1.000 | 0 | 5.83 | 0 | 80 | 10 |
| 11 | 0.243 | 3.59 | ~1.000 | 0 | 5.33 | 0 | 59 | 10 |
| 17 | 0.243 | 3.59 | ~1.000 | 0 | 5.46 | 0 | 74 | 10 |
| 20 | 0.243 | 3.59 | ~1.000 | 0 | 5.55 | 1 | 74 | 8 |
| 24 | 0.243 | 3.59 | ~1.000 | 0 | 5.19 | 0 | 82 | 8 |
| 25 | 0.243 | 3.59 | ~1.000 | 0 | 5.82 | 1 | 81 | 10 |
| 29 | 0.243 | 3.59 | ~1.000 | 0 | 5.70 | 1 | 58 | 10 |
| 35 | 0.243 | 3.59 | ~1.000 | 0 | 5.43 | 0 | 78 | 10 |
| 40 | 0.243 | 3.59 | ~1.000 | 0 | 6.17 | 0 | 48 | 10 |
| 42* | 0.243 | 3.59 | ~1.000 | 0 | 5.92 | 1 | 66 | 10 |
| 44 | 0.243 | 3.59 | ~1.000 | 0 | 5.51 | 0 | 54 | 10 |
| 45 | 0.243 | 3.59 | ~1.000 | 0 | 4.14 | 0 | 76 | 8 |
| 46 | 0.243 | 3.59 | ~1.000 | 0 | 4.66 | 1 | 51 | 8 |
| 50 | 0.243 | 3.59 | ~1.000 | 0 | 5.00 | 0 | 43 | 8 |
| 57 | 0.243 | 3.59 | ~1.000 | 0 | 5.43 | 0 | 81 | 10 |
| 64 | 0.243 | 3.59 | ~1.000 | 0 | 5.41 | 1 | 77 | 10 |
| 71 | 0.243 | 3.59 | ~1.000 | 0 | 5.55 | 0 | 73 | 8 |
| 78 | 0.243 | 3.59 | ~1.000 | 0 | 5.39 | 1 | 85 | 10 |
| 81 | 0.243 | 3.59 | ~1.000 | 0 | 5.96 | 0 | 79 | 10 |
| 82 | 0.243 | 3.59 | ~1.000 | 0 | 4.56 | 0 | 68 | 8 |
| 83 | 0.243 | 3.59 | ~1.000 | 0 | 4.10 | 1 | 78 | 8 |
| 84 | 0.243 | 3.59 | ~1.000 | 0 | 6.37 | 0 | 78 | 10 |
| 85 | 0.243 | 3.59 | ~1.000 | 0 | 5.54 | 0 | 74 | 10 |
| 86 | 0.243 | 3.59 | ~1.000 | 0 | 4.50 | 0 | 84 | 8 |
| 92 | 0.243 | 3.59 | ~1.000 | 0 | 5.14 | 0 | 74 | 8 |

*Relapsers. ε<1.
