# Supplementary material for: HCV kinetic and modeling analyses project shorter durations to cure under combined therapy with daclatasvir and asunaprevir in chronic HCV-infected patients
Source: PLoS One. 2017 Dec 7;12(12):e0187409. doi: 10.1371/journal.pone.0187409 (PMC5720697; doi:10.1371/journal.pone.0187409)
Supplement: S1 Fig — Best model fit curves are shown with black lines. Each box represent a patient (patient number in the strip above each box). The observed viral titer are represented by the dots. Data above the HCV RNA limit of quantification are shown in pink and data below the limit of detection in blue. HCV detected but not quantified is shown in green. (DOCX) [file pone.0187409.s009.docx]

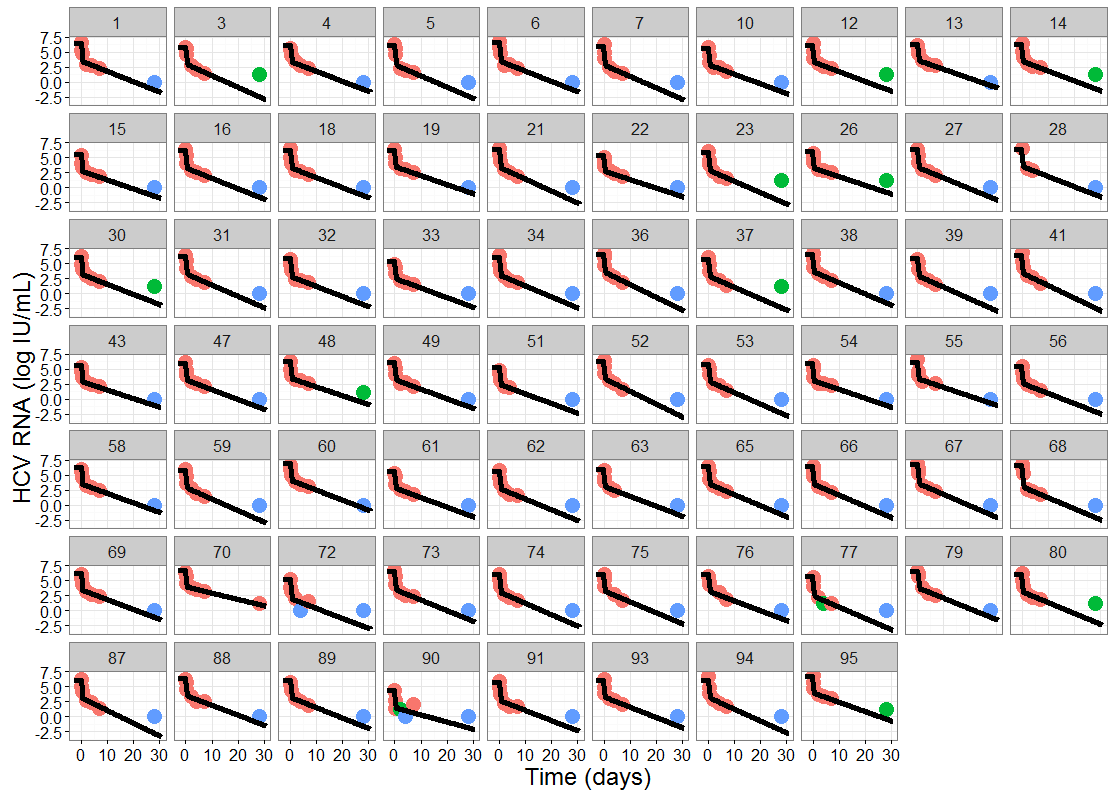


**S1 Figure:** **Individual model fits.** Best model fit curves are shown with black lines. Each box represent a patient (patient number in the strip above each box). The observed viral titer are represented by the dots. Data above the HCV RNA limit of quantification are shown in pink and data below the limit of detection in blue. HCV detected but not quantified is shown in green.
