# Supplementary material for: HCV kinetic and modeling analyses project shorter durations to cure under combined therapy with daclatasvir and asunaprevir in chronic HCV-infected patients
Source: PLoS One. 2017 Dec 7;12(12):e0187409. doi: 10.1371/journal.pone.0187409 (PMC5720697; doi:10.1371/journal.pone.0187409)
Supplement: S2 Fig — A) Visual predictive check represents the empirical percentiles (10%, 50% and 90%, green lines) and the 90% confidence intervals for these percentiles computed from 500 simulations of the observations based on the model, according to the original study design. B) Residuals plots. Observations are shown by dots and BLQ data are in red. The upper panels show population weighted residuals (PWRES) (left panel) and individual weighted residuals (IWRES) (left panels) depending on time. The lower panels PWRES (left panel) and IWRES (right panels) depending on predictions. (DOCX) [file pone.0187409.s010.docx]

A

B

**S2 Figure.** Goodness of fit plots. **A)** Visual predictive check represents the empirical percentiles (10%, 50% and 90%, green lines) and the 90% confidence intervals for these percentiles computed from 500 simulations of the observations based on the model, according to the original study design. **B)** Residuals plots. Observations are shown by dots and BLQ data are in red. The upper panels show population weighted residuals (PWRES) (left panel) and individual weighted residuals (IWRES) (left panels) depending on time. The lower panels PWRES (left panel) and IWRES (right panels) depending on predictions.
