# Supplementary material for: HCV kinetic and modeling analyses project shorter durations to cure under combined therapy with daclatasvir and asunaprevir in chronic HCV-infected patients
Source: PLoS One. 2017 Dec 7;12(12):e0187409. doi: 10.1371/journal.pone.0187409 (PMC5720697; doi:10.1371/journal.pone.0187409)
Supplement: S3 Fig — Patients with pre-treatment NS5A Y93H RAVs (Pt 75; blue line) and without (Pts 65 and 79; red curves) as described in S1 Table. Observations below the lower limit of quantification (LLOQ = 15 IU/mL) or not detected (TND) are shown with triangles whereas those above this limit are shown by circles. (DOCX) [file pone.0187409.s011.docx]

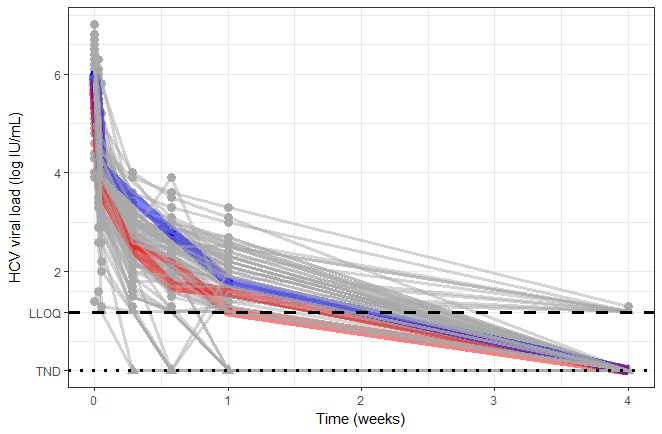


**S3 Figure: Viral kinetics in patients (n=3) with treatment- emergent resistance-associated variants, RAVs (colored lines) and without RAVs (grey lines).** Patients with pre-treatment NS5A Y93H RAVs (Pt 75; blue line) and without (Pts 65 and 79; red curves) as described in Table S1. Observations below the lower limit of quantification (LLOQ=15 IU/mL) or not detected (TND) are shown with triangles whereas those above this limit are shown by circles.
