# Supplementary material for: HCV kinetic and modeling analyses project shorter durations to cure under combined therapy with daclatasvir and asunaprevir in chronic HCV-infected patients
Source: PLoS One. 2017 Dec 7;12(12):e0187409. doi: 10.1371/journal.pone.0187409 (PMC5720697; doi:10.1371/journal.pone.0187409)
Supplement: S4 Fig — (DOCX) [file pone.0187409.s012.docx]

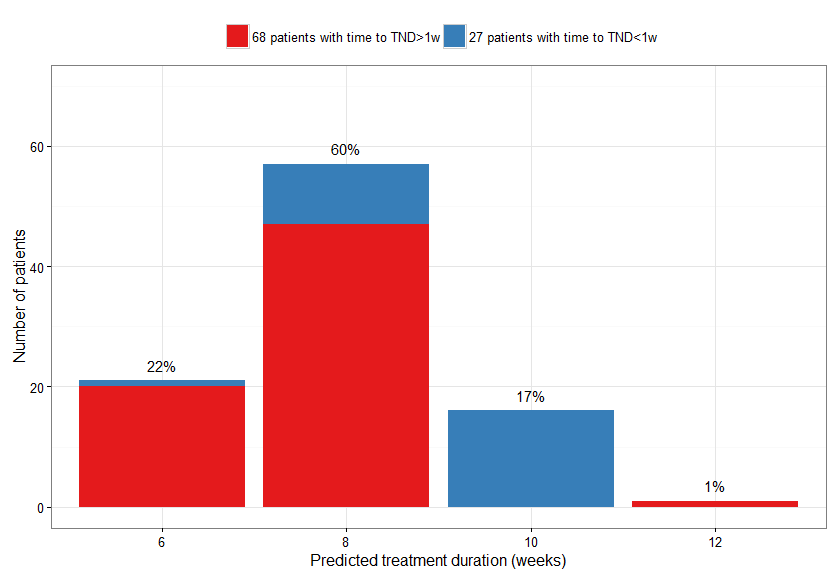


**S4 Figure.** Repartition of the predicted duration of treatment to achieve virus cure for patients with TND before 1 week (in blue) and after 1 week (in red).
